# Supplementary material for: CodonRL: Multi-Objective Codon Sequence Optimization Using Demonstration-Guided Reinforcement Learning
Source: bioRxiv. 2026 Feb 13:2026.02.12.705465. Preprint. [Version 1] doi: 10.64898/2026.02.12.705465 (PMC12918928; doi:10.64898/2026.02.12.705465)
Supplement: Supplement 1 [file NIHPP2026.02.12.705465v1-supplement-1.pdf]

bioRxiv preprint doi: https://doi.org/10.64898/2026.02.12.705465; this version posted February 14, 2026. The copyright holder for this preprint (which was not certified by peer review) is the author/funder, who has granted bioRxiv a license to display the preprint in perpetuity. It is made available under aCC-BY 4.0 International license.

A Supplementary Material

Reinforcement Learning Background

Reinforcement learning provides a principled mathematical framework for sequential decision-making under uncertainty. In this paradigm, an agent interacts with an environment by selecting actions based on observed states, receiving scalar reward signals that quantify the immediate quality of each decision [Li, 2017]. The fundamental objective is to learn a policy that maximizes the expected cumulative discounted reward over time, balancing short-term gains against long-term consequences. This framework naturally accommodates codon optimization, where each codon selection influences not only immediate sequence properties but also downstream optimization potential through effects on secondary structure and compositional constraints.

Q-learning constitutes a canonical value-based reinforcement learning algorithm that learns an action-value function representing the expected long-term reward of executing a specific action in a given state and subsequently following the optimal policy. The Q-function satisfies the Bellman optimality equation, which forms the basis for iterative learning through temporal difference updates. In deep Q-learning, the Q-function is approximated by a neural network with parameters θ, enabling application to high-dimensional state and action spaces. The network is trained by minimizing the temporal difference error between predicted Q-values and target values computed using the Bellman equation, gradually refining its estimates as more experience is accumulated.

We formulate codon optimization as a sequential decision problem amenable to reinforcement learning. In this framework, an agent learns to construct optimal mRNA sequences by iteratively selecting codons for each amino acid position, with the objective of maximizing long-term sequence quality. At each position, the agent observes a state representation encoding the partial mRNA sequence and protein context, then selects an action corresponding to a synonymous codon choice. The quality of this decision is quantified by a reward signal that evaluates structural stability and translation efficiency. A neural Q-network learns to estimate the expected cumulative reward for each possible codon selection, guiding the agent toward sequences that balance multiple competing objectives. The learning process leverages experience replay, where past decision trajectories are stored in a buffer and resampled during training to improve sample efficiency and stability. Unlike supervised learning approaches that require extensive experimental labels, reinforcement learning enables the discovery of effective optimization strategies through iterative exploration guided by computationally tractable objective functions.

Additional Related Work

Codon Optimization Methods

Codon optimization has evolved from simple frequency-matching approaches to sophisticated algorithms incorporating multiple biological constraints. Early methods focused exclusively on CAI maximization [Sharp and Li, 1987], replacing rare codons with frequently used synonymous alternatives. However, these approaches often resulted in decreased expression due to mRNA instability or translational bottlenecks.

The recognition of mRNA secondary structure's importance led to structure-aware optimization methods. LinearDesign [Zhang et al., 2023] represented a significant advance, formulating codon optimization as a joint problem of maximizing CAI while minimizing MFE through dynamic programming, achieving up to 128-fold improvement in COVID-19 vaccine antibody responses. While effective, LinearDesign employs a beam search algorithm that makes locally optimal decisions without the ability to explore radically different

bioRxiv preprint doi: https://doi.org/10.64898/2026.02.12.705465; this version posted February 14, 2026. The copyright holder for this preprint (which was not certified by peer review) is the author/funder, who has granted bioRxiv a license to display the preprint in perpetuity. It is made available under aCC-BY 4.0 International license.

solution paths.

Recent work has explored machine learning approaches to codon optimization. CodonTransformer [Fallahpour et al., 2025] employs transformer architectures with STREAM encoding trained on over 1 million DNA-protein pairs, while ICOR [Jain et al., 2023] uses bidirectional LSTM networks to capture sequential context. These supervised approaches require extensive experimental data.

Reinforcement Learning in Biological Sequence Design

Reinforcement learning (RL) has demonstrated remarkable success in biological sequence design problems. Yang et al. [2025] applied RL to regulatory element design, achieving superior performance in generating high-fitness promoters and enhancers. For protein engineering, Wang et al. [2023] developed EvoPlay using self-play RL with Monte Carlo tree search, achieving 7.8-fold bioluminescence improvement in luciferase engineering, while Sun et al. [2025] combined deep learning mutational prediction with RL for β-lactamase optimization.

However, codon optimization presents unique challenges not addressed in previous work: maintaining amino acid sequence identity, multi-objective optimization, and alleviating the computational expense of repeated RNA folding evaluations.

Efficient Transformer Architectures

Transformers have revolutionized sequence modeling but suffer from quadratic memory and computational complexity. Recent advances in efficient attention mechanisms, particularly Flash Attention [Dao et al., 2022] and Flash Attention 2 [Dao, 2023], enable linear memory scaling through tiled computation, crucial for handling long sequences in codon optimization (often exceeding 3000 nucleotides).

The application of transformers to biological sequences has shown remarkable success. ESM-1b [Rives et al., 2021] demonstrated that protein language models learn structural and functional properties from sequence alone, while the Nucleotide Transformer [Dalla-Torre et al., 2025] provides foundation models for genomic analysis. Our work builds on these foundations, adapting transformer architectures specifically for action-value estimation in Q-learning while incorporating domain-specific optimizations such as protein encoder caching.

Computational Efficiency Optimizations

Asynchronous MFE Calculation

We develop an asynchronous computation pipeline that overlaps neural network forward passes with RNA folding calculations, using a thread pool executor with $n = 4$ workers. This parallelization reduces wall-clock training time compared to synchronous evaluation.

Hierarchical LRU Caching

We employ a two-tier Least Recently Used (LRU) caching strategy with 8,192 entries for LinearFold calculations and 4,096 entries for ViennaRNA computations.

bioRxiv preprint doi: https://doi.org/10.64898/2026.02.12.705465; this version posted February 14, 2026. The copyright holder for this preprint (which was not certified by peer review) is the author/funder, who has granted bioRxiv a license to display the preprint in perpetuity. It is made available under aCC-BY 4.0 International license.

Mixed Precision Training

We leverage PyTorch's automatic mixed precision (AMP) training with the default GradScaler to accelerate computation on modern GPUs. Our implementation combines FP16 computations with FP32 master weights, achieving memory reduction while maintaining numerical stability through automatic loss scaling.

Detailed Algorithms

Algorithm 1 gives the top-level training loop. It optionally warm-starts the replay buffer using expert trajectories (Algorithm 7), selects actions with constrained ϵ-greedy decoding (Algorithm 3), computes rewards using milestone densification and terminal evaluation (Algorithms 6 and 5), and updates the Q-network by Bellman optimization with experience replay (Algorithm 4). Algorithm 2 describes inference-time multi-objective decoding using the composite scoring function. The CodonRL forward pass used by both stages is summarized in Algorithm 8.

Algorithm 1 CodonRL Training Stage

Require: Protein sequence $\mathbf{p}$, Weights $\mathbf{w}$, Trade-off λ, Episodes N
Require: Optional guide trajectory $\mathbf{g}$ from LinearDesign
Ensure: Trained Q-network Q_θ

Initialize: Networks Q_θ, Q_{θ^-}, Buffer $\mathcal{B}$, Counters
$\mathbf{H}_{\text{protein}} \leftarrow$ TransformerEncoder($\mathbf{p}$) ▷ Cached encoding
if guide $\mathbf{g}$ provided **then**
  WarmStartBuffer($\mathcal{B}, \mathbf{p}, \mathbf{g}, \mathbf{H}_{\text{protein}}$) ▷ Alg. 7
end if
$S \leftarrow 0$
for episode $e = 1$ **to** N **do**
  $\mathbf{s} \leftarrow \emptyset$
  for position $t = 1$ **to** L **do**
    $s_t \leftarrow (\mathbf{s}, t, \mathbf{H}_{\text{protein}})$
    $S \leftarrow S + 1$
    $c_t \leftarrow$ SelectCodon(Q_θ, s_t, p_t, S) ▷ Alg. 3
    $\mathbf{s} \leftarrow \mathbf{s} \oplus c_t$
    $r_t \leftarrow$ ComputeReward($\mathbf{s}, t, c_t, g_t, \lambda$) ▷ Alg. 5, 6
    $\mathcal{B} \leftarrow \mathcal{B} \cup \{(s_t, c_t, r_t, s_{t+1}, \mathbb{I}[t = L])\}$
    OptimizeBellman($Q_\theta, Q_{\theta^-}, \mathcal{B}$) ▷ Alg. 4
  end for
  TrackBestSolutions($\mathbf{s}$)
  if $e \mod \tau = 0$ **then**
    $\theta^- \leftarrow \theta$
  end if
end for
return Trained Q_θ

bioRxiv preprint doi: https://doi.org/10.64898/2026.02.12.705465; this version posted February 14, 2026. The copyright holder for this preprint (which was not certified by peer review) is the author/funder, who has granted bioRxiv a license to display the preprint in perpetuity. It is made available under aCC-BY 4.0 International license.

Algorithm 2 CodonRL Inference Stage: Multi-Objective Decoding

Require: Frozen trained Q-network Q_θ, Protein sequence $\mathbf{p}$
Require: Objective weights $\{\alpha_{\mathrm{CAI}}, \alpha_{\mathrm{CSC}}, \alpha_{\mathrm{GC}}, \alpha_{\mathrm{U}}\}$
Require: Target GC content g_{target}
Ensure: Optimized mRNA sequence $\mathbf{s}^*$

▷ Protein encoder caching: compute once, reuse across L steps
$\mathbf{H}_{\mathrm{protein}} \leftarrow$ TransformerEncoder(Embed($\mathbf{p}$) + PE($\mathbf{p}$))

$\mathbf{s} \leftarrow \emptyset$
for position $t = 1$ **to** L **do**
  $s_t \leftarrow (\mathbf{s}, t, \mathbf{H}_{\mathrm{protein}})$
  $\mathcal{A}_t \leftarrow \{c \in \mathcal{C} : \mathrm{Translate}(c) = p_t\}$ ▷ Synonymous codons, $|\mathcal{A}_t| \in [1, 6]$

  ▷ Composite scoring: learned prior + objective-specific terms
  for $c \in \mathcal{A}_t$ **do**
    $S(c, s_t) \leftarrow Q(s_t, c)$ ▷ Learned Q-value as foundation
    $S(c, s_t) \leftarrow S(c, s_t) + \alpha_{\mathrm{CAI}} \cdot \log(w_c)$ ▷ Translation efficiency
    $S(c, s_t) \leftarrow S(c, s_t) + \alpha_{\mathrm{CSC}} \cdot \log(\mathrm{CSC}_c)$ ▷ Codon stabilization coefficient
    $S(c, s_t) \leftarrow S(c, s_t) - \alpha_{\mathrm{GC}} \cdot |g(\mathbf{s} \cup \{c\}) - g_{\mathrm{target}}|$ ▷ GC content
    $S(c, s_t) \leftarrow S(c, s_t) - \alpha_{\mathrm{U}} \cdot u(c)$ ▷ Uridine minimization
  end for

  $c_t^* \leftarrow \arg\max_{c \in \mathcal{A}_t} S(c, s_t)$ ▷ Greedy selection
  $\mathbf{s} \leftarrow \mathbf{s} \oplus c_t^*$
end for

return $\mathbf{s}^* \leftarrow \mathbf{s}$

Algorithm 3 Constrained ϵ-Greedy Action Selection (SelectCodon)

Require: Q-network Q_θ, State s_t, Target amino acid $a \in \mathcal{A}$, Global step S
Ensure: Selected codon $c \in \mathcal{A}_t$, Current ϵ

$\epsilon \leftarrow \epsilon_{\mathrm{end}} + (\epsilon_{\mathrm{start}} - \epsilon_{\mathrm{end}}) \cdot \exp(-S/\kappa)$

$\mathcal{A}_t \leftarrow \{c \in \mathcal{C} : \mathrm{Translate}(c) = a\}$ ▷ Synonymous codons for amino acid a

if Uniform(0, 1) $> \epsilon$ **then**
  ▷ Exploitation: Q-value maximization with biological mask
  $\mathbf{q} \leftarrow Q_\theta(s_t)$ ▷ $\mathbf{q} \in \mathbb{R}^{64}$ for all codons
  for $c \in \mathcal{C} \setminus \mathcal{A}_t$ **do**
    $q_c \leftarrow -\infty$ ▷ Mask invalid codons
  end for
  $c \leftarrow \arg\max_c q_c$
else
  $c \leftarrow$ UniformSample($\mathcal{A}_t$) ▷ Exploration
end if

return c, ϵ

bioRxiv preprint doi: https://doi.org/10.64898/2026.02.12.705465; this version posted February 14, 2026. The copyright holder for this preprint (which was not certified by peer review) is the author/funder, who has granted bioRxiv a license to display the preprint in perpetuity. It is made available under aCC-BY 4.0 International license.

Algorithm 4 Bellman Optimization with Experience Replay (OPTIMIZEBELLMAN)

Require: Policy network Q_θ, Target network Q_{θ^-}, Buffer $\mathcal{B}$, Context $\mathbf{H}_{\text{protein}}$
Ensure: Loss value ℓ

if $|\mathcal{B}| < B$ **then**
  return $\varnothing$
end if

Sample mini-batch $\{(s_j, c_j, r_j, s'_j, d_j)\}_{j=1}^{B} \sim \mathcal{B}$

▷ Mixed precision forward pass (FP16 compute, FP32 master weights)
$\mathbf{Q} \leftarrow Q_\theta\left(\{s_j\}_{j=1}^{B}, \mathbf{H}_{\text{protein}}\right)$
$\hat{Q}_j \leftarrow Q_{j,c_j}$ for all $j \in [B]$

for $j = 1$ **to** B **do** ▷ Temporal difference targets
  if $d_j = 1$ **or** $s'_j = \varnothing$ **then**
    $y_j \leftarrow r_j$
  else
    $\mathbf{q}'_j \leftarrow Q_{\theta^-}(s'_j, \mathbf{H}_{\text{protein}})$
    $y_j \leftarrow r_j + \gamma \cdot \max_c q'_{j,c}$
  end if
end for

$\ell \leftarrow \frac{1}{B} \sum_{j=1}^{B} \mathcal{L}_{\text{Huber}}(\hat{Q}_j, y_j)$
$\theta \leftarrow \theta - \alpha \nabla_\theta \ell$ ▷ Gradient update with automatic loss scaling

return ℓ

Algorithm 5 Multi-Objective Reward Computation (COMPUTEREWARD)

Require: mRNA sequence $\mathbf{s}$, Trade-off λ, Adaptiveness $\mathbf{w}$
Ensure: Total reward $\mathcal{R}_{\text{total}}$, MFE value

▷ Thermodynamically rigorous MFE via ViennaRNA
$\text{MFE}(\mathbf{s}) \leftarrow \text{VIENNARNA}(\mathbf{s})$

▷ Log-sum of relative adaptiveness
$\sum_{i=1}^{L} \log(w_i) \leftarrow \sum_{i=1}^{L} \log\left(\frac{f_{c_i}}{\max_{c \in \text{syn}(i)} f_c}\right)$

▷ Combined objective balancing stability and translation efficiency
$\mathcal{R}_{\text{total}}(\mathbf{s}) \leftarrow -\left[\text{MFE}(\mathbf{s}) - \lambda \sum_{i=1}^{L} \log(w_i)\right]$

return $\mathcal{R}_{\text{total}}, \text{MFE}(\mathbf{s})$

bioRxiv preprint doi: https://doi.org/10.64898/2026.02.12.705465; this version posted February 14, 2026. The copyright holder for this preprint (which was not certified by peer review) is the author/funder, who has granted bioRxiv a license to display the preprint in perpetuity. It is made available under aCC-BY 4.0 International license.

Algorithm 6 Milestone Reward for Reward Densification (MILESTONEREWARD)

Require: Partial mRNA $\mathbf{s}_{1:k}$, Trade-off λ, Adaptiveness $\mathbf{w}$
Ensure: Milestone reward $\mathcal{R}_k^{\text{guide}}$

$n \leftarrow |\mathbf{s}_{1:k}|$ ▷ Current sequence length in nucleotides

if $n < 4$ **then**
  return 0
end if

▷ LinearFold for $O(L)$ complexity at intermediate milestones
$\text{MFE}_{1:k} \leftarrow \text{LINEARFOLD}(\mathbf{s}_{1:k})$

▷ Partial CAI computation
$\text{CAI}_{1:k} \leftarrow \exp\left(\frac{1}{k}\sum_{i=1}^{k}\log w_i\right)$

▷ Per-position normalized milestone objective
$\mathcal{R}_k^{\text{guide}} \leftarrow -\left[\frac{\text{MFE}_{1:k}}{k} - \lambda\log(\text{CAI}_{1:k})\right]$

return $\mathcal{R}_k^{\text{guide}}$

Algorithm 7 Replay Buffer Warm-Start with Expert Demonstrations (WARMSTARTBUFFER)

Require: Buffer $\mathcal{B}$, Protein $\mathbf{p}$, Guide sequence $\mathbf{g}$, Context $\mathbf{H}_{\text{protein}}$

▷ Compute final reward for expert trajectory
$\mathcal{R}_{\text{final}}, \_ \leftarrow \text{COMPUTEREWARD}(\mathbf{g}, \lambda)$

$\mathbf{s} \leftarrow \emptyset$
for $t = 1$ **to** L **do**
  $s_t \leftarrow (\mathbf{s}, t, \mathbf{H}_{\text{protein}})$
  $c_t \leftarrow g_t$ ▷ Expert action from guide sequence
  $\mathbf{s} \leftarrow \mathbf{s} \oplus c_t$

  $r_t \leftarrow \beta$ ▷ Auxiliary imitation bonus
  if $t \in \Phi$ **then**
    $r_t \leftarrow r_t + \beta_k \cdot \text{MILESTONEREWARD}(\mathbf{s}, \lambda)$
  end if
  if $t = L$ **then**
    $r_t \leftarrow r_t + \mathcal{R}_{\text{final}}$
  end if
  $s_{t+1} \leftarrow (\mathbf{s}, t+1, \mathbf{H}_{\text{protein}})$ **if** $t < L$ **else** $\varnothing$

  ▷ Pre-populate buffer: $\mathcal{B}_0 = \{(s_t^{\text{guide}}, a_t^{\text{guide}}, r_t^{\text{guide}} + \beta, s_{t+1}^{\text{guide}})\}$
  $\mathcal{B} \leftarrow \mathcal{B} \cup \{(s_t, c_t, r_t, s_{t+1}, \mathbb{I}[t = L])\}$
end for

bioRxiv preprint doi: https://doi.org/10.64898/2026.02.12.705465; this version posted February 14, 2026. The copyright holder for this preprint (which was not certified by peer review) is the author/funder, who has granted bioRxiv a license to display the preprint in perpetuity. It is made available under aCC-BY 4.0 International license.

Algorithm 8 Flash Attention-based Q-Network Forward Pass (CodonRLForward)

Require: Partial mRNA $\mathbf{s}$, Position t, Cached protein memory $\mathbf{H}_{\text{protein}} \in \mathbb{R}^{L\times d}$
Ensure: Q-values $Q(a_t \mid t) \in \mathbb{R}^{64}$ for all 64 codons

▷ mRNA decoder embedding
$\mathbf{E}_{\text{mRNA}} \leftarrow \text{CodonEmbed}(\mathbf{s}) \cdot \sqrt{d}$
$\mathbf{E}_{\text{mRNA}} \leftarrow \mathbf{E}_{\text{mRNA}} + \text{PE}(t)$

▷ Causal transformer decoder with Flash Attention
$\mathbf{M}_{\text{causal}} \leftarrow \text{CausalMask}(t)$
$\mathbf{H}_{\text{mRNA}} \leftarrow \text{TransformerDecoder}(\mathbf{E}_{\text{mRNA}}, \mathbf{H}_{\text{protein}}, \mathbf{M}_{\text{causal}})$

▷ Position-aware Q-value via cross-attention
$\mathbf{h}_t^{\text{mRNA}} \leftarrow \mathbf{H}_{\text{mRNA}}[t, :]$

▷ Cross-attention to local and global protein context
$\mathbf{z}_t \leftarrow \text{CrossAttention}\left(\mathbf{h}_t^{\text{mRNA}}, \mathbf{H}_{\text{protein}}[t], \mathbf{H}_{\text{protein}}\right)$

▷ MLP head for action-value estimation
$Q(a_t \mid t) \leftarrow \text{MLP}(\mathbf{z}_t)$

return $Q(a_t \mid t)$

bioRxiv preprint doi: https://doi.org/10.64898/2026.02.12.705465; this version posted February 14, 2026. The copyright holder for this preprint (which was not certified by peer review) is the author/funder, who has granted bioRxiv a license to display the preprint in perpetuity. It is made available under aCC-BY 4.0 International license.

Runtime Comparison between ViennaRNA and LinearFold

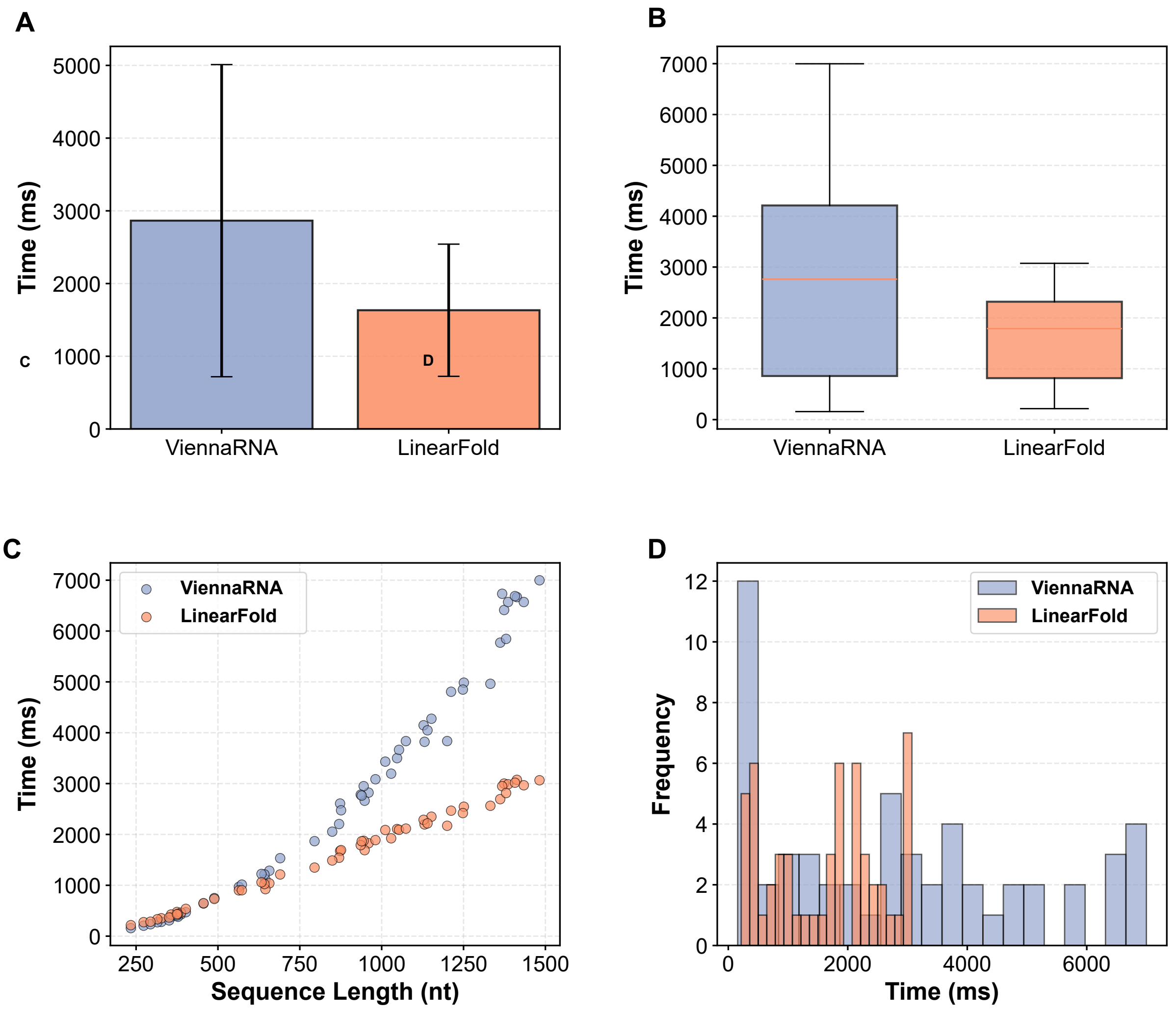

Figure 7: **Comprehensive runtime comparison between ViennaRNA and LinearFold for MFE computation. (A)** Mean runtime comparison. Bar plot showing the average computation time for minimum free energy (MFE) prediction using ViennaRNA (2864.90 $\pm$ 2146.16 ms, blue) and LinearFold (1633.05 $\pm$ 908.95 ms, orange). Error bars represent standard deviation ($n = 55$ sequences). **(B)** Runtime distribution. Box plots illustrating the distribution of computation times for both methods. Boxes represent the interquartile range (IQR), horizontal lines indicate medians (ViennaRNA: 2763.15 ms; LinearFold: 1789.36 ms), and red diamonds denote mean values. **(C)** Runtime vs. sequence length. Scatter plot showing the relationship between RNA sequence length (234–1482 nt) and computation time for both ViennaRNA (blue) and LinearFold (orange). Each dot represents one sequence. **(D)** Runtime frequency distribution. Overlapping histograms displaying the frequency distribution of computation times across all test sequences. LinearFold achieves 1.75× speedup over ViennaRNA with 57.7% lower variance (std: 908.95 ms vs. 2146.16 ms).
